# Task demands modulate decision and eye movement responses in the chimeric face test: examining the right hemisphere processing account

**DOI:** 10.3389/fpsyg.2014.00229

**Published:** 2014-03-20

**Authors:** Jason C. Coronel, Kara D. Federmeier

**Affiliations:** ^1^Annenberg School for Communication, University of PennsylvaniaPhiladelphia, PA, USA; ^2^Department of Psychology, Program in Neurosciences, and Beckman Institute for Advanced Science and Technology, University of IllinoisChampaign, IL, USA

**Keywords:** chimeric face test, right hemisphere processing account, scanning bias, eye movements, lateralization of emotion

## Abstract

A large and growing body of work, conducted in both brain-intact and brain-damaged populations, has used the free viewing chimeric face test as a measure of hemispheric dominance for the extraction of emotional information from faces. These studies generally show that normal right-handed individuals tend to perceive chimeric faces as more emotional if the emotional expression is presented on the half of the face to the viewer's left (“left hemiface”). However, the mechanisms underlying this lateralized bias remain unclear. Here, we examine the extent to which this bias is driven by right hemisphere processing advantages vs. default scanning biases in a unique way—by changing task demands. In particular, we compare the original task with one in which right-hemisphere-biased processing cannot provide a decision advantage. Our behavioral and eye movement data are inconsistent with the predictions of a default scanning bias account and support the idea that the left hemiface bias found in the chimeric face test is largely due to strategic use of right hemisphere processing mechanisms.

## Introduction

Hemispheric specialization is a fundamental feature of how the human brain is organized for cognition. Over the past several decades, research has shown that each hemisphere has its own set of capacities and specializations in a variety of domains, including language, spatial processing, and emotional processing (for reviews, see Gazzaniga, 1995; Hervé et al., 2013). Such specializations have been argued to increase the information processing capacity of the brain (Friedman and Polson, 1981; Rogers, 2000) and to allow multiple processing strategies that address computational tradeoffs (e.g., Banich and Belger, 1990; Kosslyn et al., 1992; Federmeier, 2007). However, it remains unclear whether, and, if so, how, the brain can strategically deploy these strategies. Is hemispheric dominance, the tendency for one hemisphere to assume control of processing, fixed for certain forms of information, at least within a given individual? Or are there strategies, such as the deployment of attention to contralateral information, that can be used to flexibly recruit lateralized processing mechanisms for the task at hand (e.g., Levy and Trevarthen, 1976; Hellige and Michimata, 1989; Weissman and Banich, 2000)?

**Figure 1:**
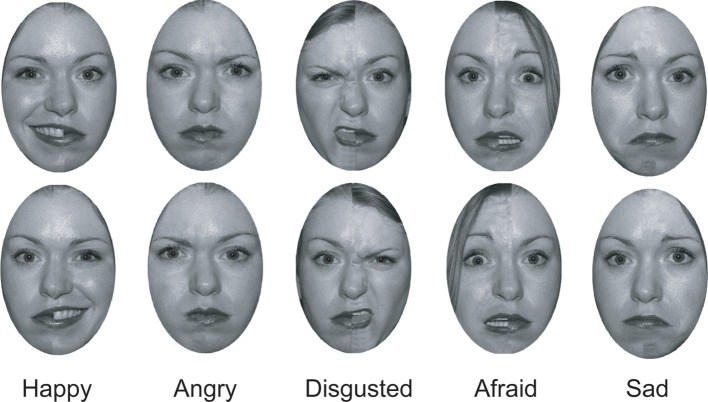
**Examples of the chimeric faces used in this study**.

One robust but still incompletely understood metric of hemispheric dominance for face/emotion processing comes from the chimeric face test. This test involves the presentation of chimeric faces, which are vertically split composites of what is usually the same person's face displaying a different expression on each half. For example, in the original version of the paradigm, one side of the hemiface conveys a positive emotional expression (i.e., a person smiling) and the other side a neutral expression (Levy et al., 1983). A chimeric face and its mirror image are presented one above the other (see Figure 1, far left), and participants are instructed to indicate which of the two chimeric faces looks happier. Even though the two chimeric faces contain the same information, as one is just a mirror image of the other, neurologically intact right-handed individuals have a tendency to pick the face in which the emotional expression is conveyed on the viewer's left side (the “left hemiface”; for a review and meta-analysis, see Voyer et al., 2012). This left hemiface bias is robust and has been replicated using samples from different cultures (Vaid and Singh, 1989) and age groups (bias emerges as early as 5 years old: Failla et al., 2003) and in versions of the test that make significant modifications to the stimuli. For instance, the use of negative emotional expressions, inverted faces, or cartoon faces may reduce, but do not abolish, the bias (Hoptman and Levy, 1988; Christman and Hackworth, 1993; Luh, 1998; Butler and Harvey, 2005; Parente and Tommasi, 2008; Bourne, 2010, 2011). Furthermore, this left hemiface bias for chimeric faces extends beyond emotional expressions. Other versions of the paradigm in which the differences between the left and right side of the faces are based on age, sex, or attractiveness have also been shown to elicit a left hemiface decision bias (Luh et al., 1991; Burt and Perrett, 1997). These biases are reflected in reaction times as well as decision proportions: participants are generally faster to respond on trials in which they pick the left hemiface than those in which they pick the right hemiface (Bourne, 2008).

A prevailing explanation for the bias observed in the chimeric face test is that the results reflect a right hemisphere dominance for extracting information from faces, perhaps especially emotional information (Voyer et al., 2012). Some of the strongest evidence supporting this claim comes from studies using brain-damaged populations. In particular, patients with unilateral right hemisphere lesions show a decreased left hemiface bias (Kucharska-Pietura and David, 2003). In contrast, patients with unilateral left hemisphere lesions show an increased left hemiface bias (meta-analysis by Voyer et al., 2012); one interpretation is that this results from a decrease in competition from the left hemisphere. Thus, the hypothesis is that right hemisphere superiority for aspects of face (and/or emotion) processing allows better extraction of decision-critical information from chimeric faces that contain that information in the left visual field.

Although the pattern observed on the chimeric face test has often been interpreted as a stimulus-driven perceptual bias linked to hemispheric specialization, other factors have been found to modulate performance on the test. Levy et al. (1983) found stable and reliable patterns of individual differences in degree of bias on the test, even within right-handed individuals (who would be presumed to have similar patterns of hemispheric specialization), and linked these to global attentional biases. Others have suggested that the left hemiface bias may be importantly driven by well-practiced directional scanning biases, and thus not as reflective of hemispheric specialization as is typically assumed (cf. Bryden, 1966; Vaid and Singh, 1989; Heath et al., 2005). That is, the extent to which an individual has a learned tendency—for example, based on reading experience with a particular language—to scan from left to right (or vice versa) in evaluating stimuli can privilege processing in one visual field. Vaid and Singh (1989) examined this possibility by measuring performance on a happy/neutral chimeric face test using three groups whose native languages differed in their scanning patterns: Hindi readers, who scan from left to right, Arabic readers, who scan from right to left, and Urdu readers, who scan from right to left, but who also had exposure to Hindi and thus were classified as bidirectional. Consistent with a directional scanning account, this study found greater left hemiface bias in Hindi readers compared to the other two groups (Vaid and Singh, 1989).

Another study that tracked eye movements as participants viewed (male/female) chimeric face pairs also showed that decision biases were related to fixation patterns in a manner that is consistent with the directional scanning account (Butler et al., 2005). In particular, this study found that participants were more likely to show a left hemiface bias when they spent more time fixating on the left side of the chimeric face. However, a follow-up study by the same group restricted scanning time by displaying the chimeric faces for only 100 ms. They found that right-handed participants still showed a left hemiface bias, suggesting that scanning biases may not be necessary for eliciting the effect (Butler and Harvey, 2006).

Thus, it remains unclear precisely what mechanisms underlie the oft-observed left hemiface bias. In the current study, we further examine the mechanisms at work in the elicitation of this bias by examining the effect that task demands have for eye gaze patterns and choice behavior in the chimeric faces test. Prior work in the literature, as described above, has largely focused on varying the types of stimuli or the duration of stimulus presentation used in the test. No study, to our knowledge, has systematically examined how changes in task demands can affect performance on the test. Almost all studies have used relatively similar tasks in which participants' judgments about the faces are directly related to the aspects of the face that are manipulated across the two halves. For instance, studies that manipulate emotional expression ask participants to identify which of the chimeric faces look happier, sadder, angrier, etc. Studies that manipulate sex ask participants to identify which of the chimeric faces look male or female. Importantly, these manipulated characteristics are ones for which the right hemisphere might have a processing advantage (Demaree et al., 2005; Hu et al., 2013).

Critically, in this study, we compare the standard chimeric faces emotion judgment task with a task for which information from the left hemiface would not be expected to provide decision-critical information (an “original/mirror image” judgment task, described below). We monitored eye movements to be able to look simultaneously at scanning patterns and behavioral decisions. If the tendency to look at the left hemiface is a default bias, created by reading experience or scanning patterns learned for faces, then gaze patterns should be similar in the two tasks. If, then, these patterns drive the decision bias, we should see a similar decision bias in the two tasks. This outcome would provide strong support for a scanning bias account of performance on the chimeric faces task. However, if a left hemiface bias arises because of right hemisphere specialization for extracting certain types of information, such as emotion, from faces, then we would expect a reduction or elimination of the left hemiface bias in our alternative task compared to the standard task—despite using the same subject population, the same stimuli, and the same general paradigm. Finally, if gaze patterns differ across the tasks, then this would support the view that participants may use gaze (and, by inference, attention) to strategically recruit specialized hemispheric resources to meet task demands.

## Methods

### Participants

We recruited 66 individuals from the University of Illinois to take part in the study in exchange for monetary compensation. All participants, by self-report, were native English speakers. Eight participants were excluded, as they were classified as either left handed or ambidextrous as assessed by the Edinburgh Inventory (Oldfield, 1971). The remaining 58 participants were right-handed. Twenty-nine participants [16 females, average age = 21 (range = 18–29), handedness score = 79.3] were randomly assigned to Task 1 (emotion judgment task) and 29 participants [19 females, average age = 23 (range = 19–34), handedness score = 78.4] were assigned to Task 2 (original face judgment task).

### Stimuli

We constructed chimeric faces from a set of photos of normal faces obtained from the NimStim database (Tottenham et al., 2009). We retrieved six types of emotive faces from this set: neutral, angry, happy, disgusted, sad, and fearful. Faces had been previously normed by the Tottenham et al. group to ensure that a majority of individuals correctly perceived each face as conveying a particular emotional expression.

Using Adobe Photoshop, we converted each face into gray scale and removed extra-facial details, such the head hair and ears. We created chimeric faces for each emotion category by splitting each face in half and combining the left half of a face displaying emotion with the right half of the face of the same individual displaying a neutral expression. We smoothed the area where the two halves of the faces met in order to give the appearance of a continuous face. A mirror image of each face was then produced and the faces were placed one above another, with the location of the original (e.g., emotion on the left hemiface) and the mirror-image counterbalanced. There were five categories of chimeric faces: happy-neutral, disgust-neutral, angry-neutral, sad-neutral, and fearful-neutral. In each category, there were a total of 18 unique faces (nine men, nine women). Thus, there were a total of 90 chimeric face pairs (see Figure 1).

### Procedure

Participants were tested individually in a quiet room, where they were seated 100 cm away from a 22-in. Cornerstone P1750 monitor (resolution 1024 × 768), with a refresh rate of 60 Hz. Before the experiment began, the desktop-mounted SR Research EyeLink 1000 eye tracker was calibrated for each subject with a 9-point calibration system. A chin rest was used to reduce head movements. Drift correction was done at the beginning of each trial. Recordings were monocular, taken from the right eye.

Participants that were randomly assigned to Task 1 were given a standard chimeric face test. A single trial began with the 2 s presentation of a sentence asking, “Which face looks happier/sadder/angrier/more fearful/more disgusted?” This screen was then replaced by a drift-check target. In order to advance from this target, participants had to fixate accurately on the target while pressing the advance button on a handheld controller. They were then presented with two chimeric faces. One chimeric face was presented on the top half of the screen and its mirror image was presented on the bottom half. The top/bottom location of each chimeric face (e.g., emotion on the left hemiface) was counterbalanced across participants. Participants pressed one of two buttons on a handheld controller to indicate which of the two faces was more expressive of the cued emotion; they were told to try to make their judgments in less than 10 s. In addition, they were told that the faces would be on the screen for a minimum of 10 s. This meant that if they pressed the button in less than 10 s, the faces would still remain on the screen until 10 s from the onset of the stimuli had transpired. They were then presented again with a drift check target that indicated the start of a new trial.

For Task 2, participants were given a different set of instructions. Prior to the start of the study, we showed participants pairs of chimeric faces. We told them that in order to construct the pair of chimeric faces, we first had to create an “original chimeric face” by combining two halves of photographs of the same person (e.g., left half from a photo of a person displaying happiness and right half from a photo of the same person displaying a neutral expression, or vice versa). We explained that the other chimeric face was then a mirror image of that “original chimeric face.” We told participants that for each pair, they should try to determine which chimeric face was the “original” one. We expected that these instructions would motivate participants to carefully examine the faces and extract perceptual information to try to use as the basis for making these judgments; indeed, as described below, their reaction time data makes clear that they took the task seriously. However, we did not expect that participants would actually be able to accurately detect the “original chimeric face,” as we don't believe there are any perceptual signatures of which side of the face the emotional (or neutral) expression was originally on. The aim was for this task to share similar task demands as the standard chimeric faces test (i.e., participants need to study the faces and make a choice decision), but under circumstances in which we can be certain that right hemisphere specializations for face processing could not provide any useful information.

Individual trials followed the same structure as in Task 1: drift check, presentation of chimeric face pairs for 10 s, and participant decision, signaled with a button press response. The same pairs of chimeric faces were presented in Task 1 and Task 2. For analysis purposes, we averaged across emotional expression to maximize our power to see task differences, since past work has shown overall left hemiface biases across types of emotional expression (Christman and Hackworth, 1993; Bourne, 2010) and since responses to different emotional expressions were not of theoretical interest here.

## Results

### Behavioral judgments

We analyzed the data in a logistic mixed effects model with a binomial link function, with Task (coded as “1” for Task 1 and “−1” for Task 2) as a fixed effect and participants and items (i.e., each chimeric face trial) as random effects (see Table 1). The dependent variable was whether the participant picked the chimeric face in which the emotional expression was presented on the left side (the left hemiface). This model revealed a main effect of Task (*z* = 5.83, *p* < 0.001): Participants were more likely to pick the left hemiface in Task 1 than in Task 2.

**Table 1:** **Influence of Task on Behavioral Judgments**.

	**Estimate**	***SE***	***z*-value**	***Pr*(>|*z*|)**
**FIXED EFFECTS**				
(Intercept)	0.71	0.12	5.67	<0.001
Task	0.70	0.12	5.83	<**0.001**

*Bold values correspond to statistically significant*.

Next, following the analytical strategy of prior studies (Levy et al., 1983; for a review, see Voyer et al., 2012), we created a lateralization quotient (LQ) score in order to estimate the hemiface judgment bias of each participant. To calculate the LQ score, we obtained the number of times an individual selected the face in which the emotional expression was located in the right hemiface and subtracted from this value the number of times the participant selected the face in which the emotional expression was in the left hemiface. This value was then divided by the total number of trials (i.e., 90). Thus, an LQ greater than zero indicates a right hemiface bias, a score of zero indicates no bias, and a score less than zero indicates a left hemiface bias.

**Figure 2:**
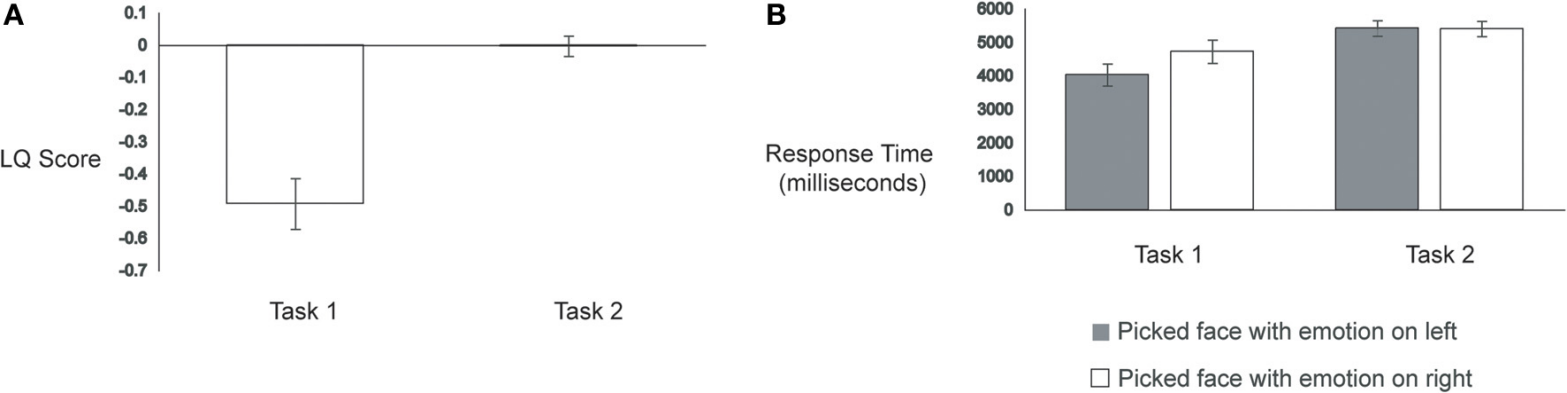
**(A)** Laterality Quotient Scores and **(B)** Response Times across the two tasks.

Consistent with previous studies, participants in Task 1 displayed a robust left hemiface bias (mean LQ score = −0.49), *t*_(28)_ = −6.23, *p* < 0.001. Strikingly, participants in Task 2 showed a much lower LQ score (mean = −0.003), *t*_(56)_ = −5.75, *p* < 0.001, which was not reliably different from zero, *t*_(28)_ = −0.10, *p* = 0.92. See Figure 2A. Thus, as expected, participants in Task 2 were not able to detect reliably the original chimeric face; accurate detection would have yielded a left hemiface bias, since all chimeric faces were originally constructed using the left half of emotion-conveying faces.

### Response times

Prior work using happy/neutral chimeric faces has shown that participants respond more quickly on trials wherein they show a left hemiface bias compared to trials wherein they show a right hemiface bias (Bourne, 2008). To test for this pattern in our data, we calculated response times from the onset of the chimeric face pairs to the participant's button press response. Trials with response times that were two standard deviations above each participant's average response time were removed, yielding an average loss of 4% of trials for Task 1 and 3% of trials for Task 2 (these trials were also removed from the LQ score and eye-movement analyses). A two-factor ANOVA with Task (Task 1, Task 2) as a between-subjects factor and Face Judgment (picked left hemiface, picked right hemiface) as a within-subjects factor revealed a main effect of Task, with Task 1 eliciting faster response times (mean = 4381 ms) than Task 2 (mean = 5415 ms), *F*_(1, 56)_ = 6.71, *p* = 0.01, and a main effect of Face Judgment, wherein trials in which participants showed a left hemiface bias (mean = 4729 ms) elicited faster response times than trials in which participants displayed a right hemiface bias (mean = 5068 ms), *F*_(1, 56)_ = 14.03, *p* < 0.001. These main effects, however, were moderated by a significant Task × Face Judgment interaction, *F*_(1, 56)_ = 15.77, *p* < 0.001.

Follow-up analyses revealed that the faster response times to left hemiface-biased trials (mean = 4032 ms) compared to right hemiface-biased trials (mean = 4730 ms) occurred only for Task 1, *t*_(28)_ = 4.42, *p* < 0.001. In Task 2, response times to left hemiface-biased trials (mean = 5425 ms) were indistinguishable from those to right hemiface-biased trials (mean = 5405 ms), *t*_(28)_ = −0.23, *p* = 0.82 (see Figure 2B).

### Gaze patterns

**Figure 3:**
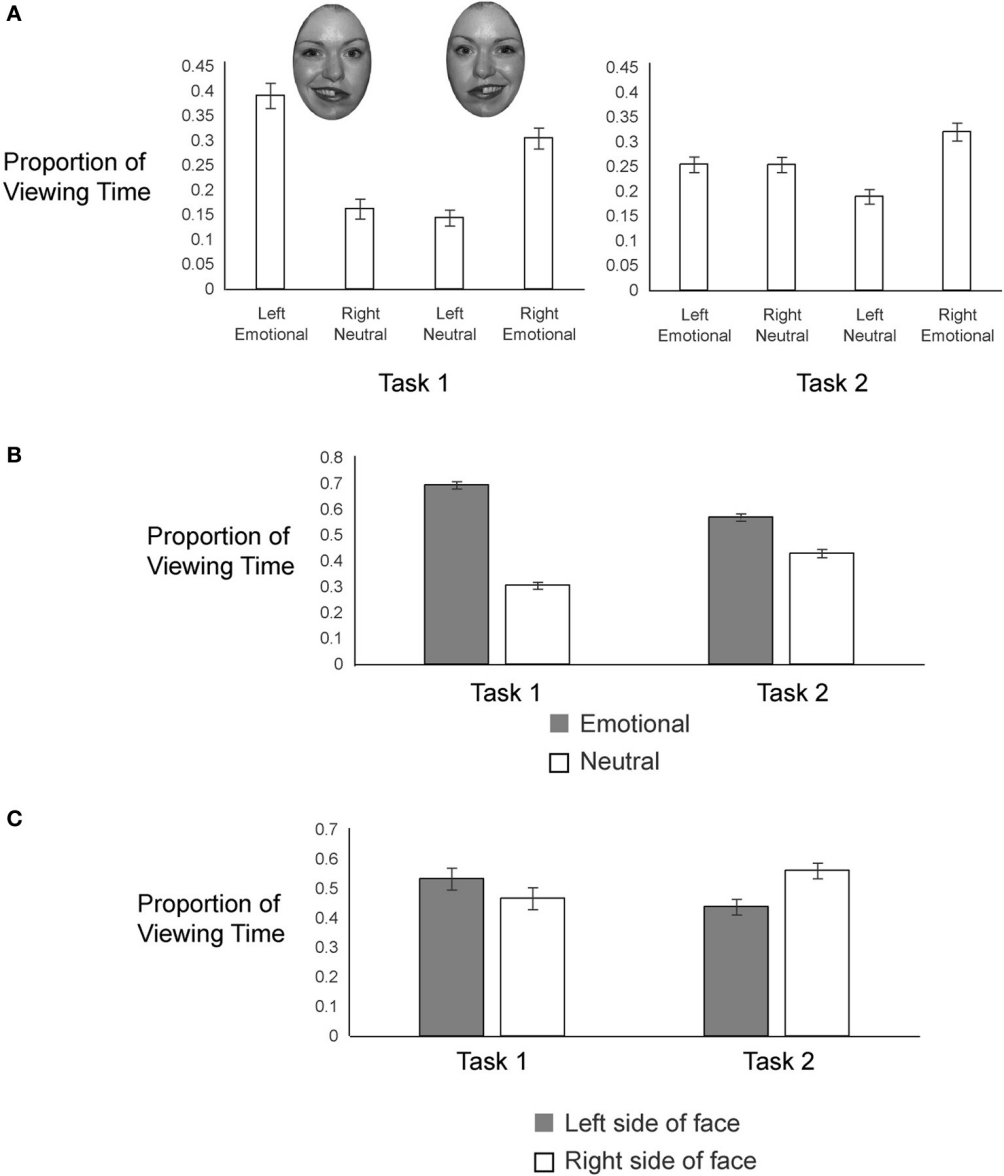
**Proportion of Viewing Time to (A) Four Regions of Interest, (B) Emotional/Neutral and (C) Left/Right Sides of the Faces Across Tasks**.

To examine gaze patterns, we created four regions of interest encompassing the two hemifaces of each of the two chimeric images: emotional expression on the left side, neutral expression on the right side (from one chimeric face), emotional expression on the right side, neutral expression on the left side (from the other chimeric face). We obtained the proportion of viewing time a participant spent on each of the four regions of interest by determining the duration of fixations to a given interest area and dividing that value by the combined duration of fixations for all four regions of interest. This measure was calculated for each trial, beginning from the onset of the chimeric faces and terminating when participants pressed the button to register their choice. Proportion of looks to each side of each chimeric face in each task is plotted in Figure 3.

In both tasks, participants gazed more overall at the emotional sides of the faces (69% in Task 1 and 57% in Task 2) than at the neutral sides of the faces (Figure 3B), but this bias to look at the emotional half faces was greater in Task 1 [*t*_(56)_ = −5.90, *p* < 0.001]. In Task 1, participants viewed the left halves of the faces more (53%) than the right halves of the faces, whereas in Task 2, participants were biased toward looking at the right halves of the faces (gaze proportion to left = 44%) (Figure 3C); this task difference in lateralized gaze preference was significant [*t*_(56)_ = −2.07, *p* = 0.04].

**Figure 4:**
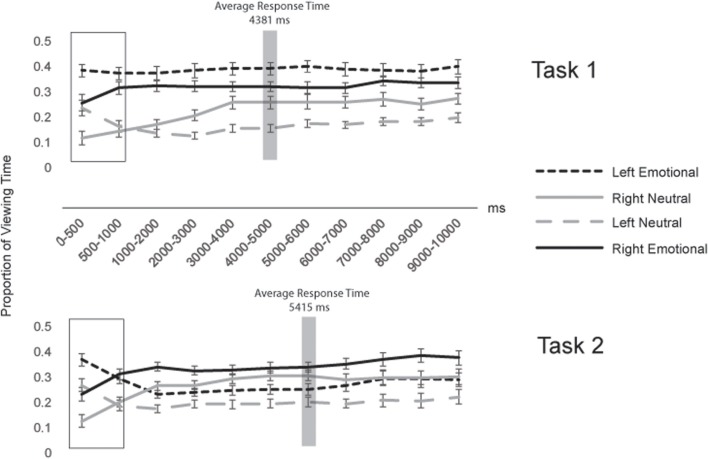
**Proportion of Viewing Time to Four Regions of Interest in 1000 ms Time Bins**.

In addition, we examined how gaze patterns developed over time. Figure 4 shows the proportion of viewing time directed to each region of interest across the entire 10 s that the faces were on the screen, for successive 1000 ms time bins. Viewing proportions were calculated separately in each bin. To capture early gaze patterns, we also split the first time bin in half, looking separately at 0–500 ms and 500–1000 ms. In the first 500 ms after stimulus onset (see box in Figure 4), participants in both tasks showed similar preferences to view left over right hemifaces and emotional over neutral hemifaces, resulting in the highest proportion of gaze being directed to the left emotional hemiface [38% in Task 1 and 37% in Task 2; these proportions did not differ by Task: *t*_(56)_ = 0.40, *p* = 0.69]. However, as can be seen in Figure 4, after the first 500 ms, gaze patterns diverged across task, such that by the 1000–2000 ms time bin, they stabilized at the pattern characterized by the overall gaze proportions (wherein Task 1 participants continued to gaze most at the left emotional hemiface, but Task 2 participants switched to gaze most at the right emotional hemiface). Importantly, this pattern was sustained up to and beyond the response times for both tasks (meaning that overall gaze patterns were not skewed by different cutoff times based on the response time difference across tasks) (see Figure 4).

### Gaze patterns and choice behavior

**Figure 5:**
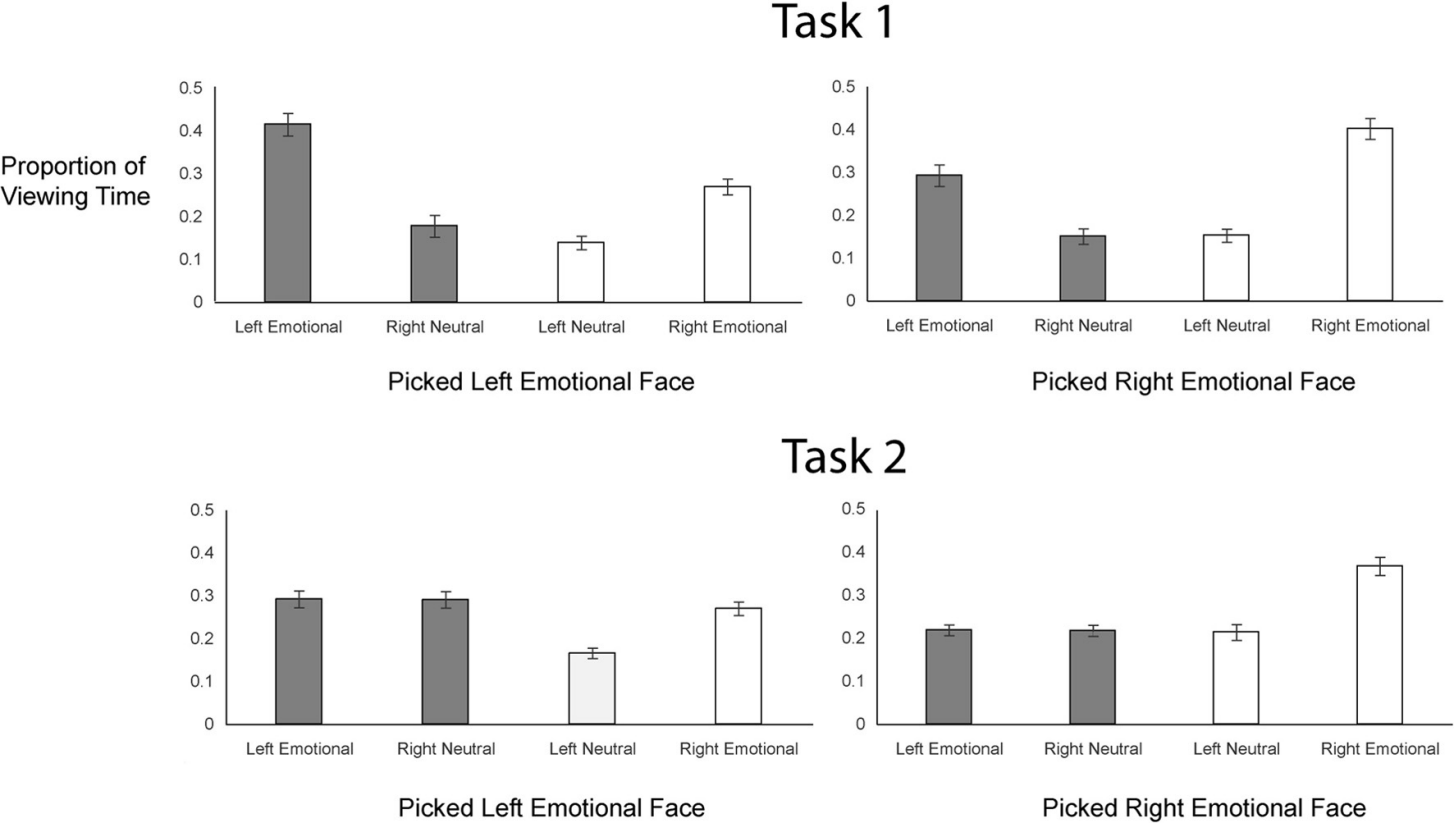
**Gaze Patterns as a Function of Task and Behavioral Choice**.

Critically, the scanning bias account claims that gaze patterns prior to the behavioral decision should predict choice outcomes and should do so similarly across task. Figure 5 shows gaze patterns as a function of task and behavioral choice. To assess how gaze patterns were related to choice behavior as a function of task, we first tested the hypothesis that a general bias to gaze at the left side of the chimeric faces predicted a left emotional hemiface bias in choice behavior. We analyzed the data in a logistic mixed effects model with a binomial link function, with Task (coded as “1” for Task 1 and “−1” for Task 2) and Left Proportion (i.e., mean-centered proportion of time spent looking on the left side of the faces) as fixed effects, and participants and items as random effects (see Table 2). This model revealed a main effect of Task (*z* = 5.27, *p* < 0.001): as already shown, participants were more likely to pick the left hemiface in Task 1 than in Task 2. There was also a main effect of Left Proportion (*z* = 9.33, *p* < 0.001) as participants were more likely to pick the left hemiface as they spent a greater amount of time looking at the left side of the faces. Finally, there was a significant Task × Left Proportion interaction (*z* = 4.02, *p* < 0.001): an increase in looking at the left side of the face had a greater impact on picking the left hemiface in Task 1 than in Task 2. (The same analysis done for gaze patterns between 0 and 500 ms revealed no relationship between *initial* gaze and choice behavior in either task; see Table S2 in Supplementary Materials Section).

**Table 2:** **Influence of Task and Gaze Patterns Directed to the Left Side on Behavioral Judgments**.

	**Estimate**	***SE***	***z*-value**	***Pr*(>|*z*|)**
**FIXED EFFECTS**				
(Intercept)	0.67	0.12	5.71	<0.001
Task	0.61	0.12	5.27	<**0.001**
Left proportion	1.8	0.19	9.33	<**0.001**
Task ^*^ left proportion	0.77	0.19	4.02	<**0.001**

*Bold values correspond to statistically significant*.

We further explored this interaction by examining the relationship of gaze to each of the four interest areas on choice behavior (see Table S1 in Supplementary Materials Section). For each region, we conducted analyses using a logistic mixed effects model with a binomial link function, with Task (coded as “1” for Task 1 and “−1” for Task 2) and proportion (centered on the mean) as fixed effects, and participants and items as random effects. The main effect of Task, present in each analysis, has already been described. There were main effects of Left Emotional (*z* = 15.77, *p* < 0.001) and Right Neutral (*z* = 11.88, *p* < 0.001) proportions (i.e., interest areas that constitute the left hemiface, with emotion on the left and a neutral expression on the right). More gaze in either of these interest regions was associated with a greater likelihood of picking that left hemiface. However, choice behavior was more driven by looks to the Left Emotional hemiface in Task 1 (vs. Task 2; *z* = 2.80, *p* < 0.01), whereas it was more driven by looks to the Right Neutral hemiface in Task 2 (vs. Task 1; *z* = −3.73, *p* < 0.001). There were also main effects of Left Neutral (*z* = −8.32, *p* < 0.001) and Right Emotional (*z* = −20.68, *p* < 0.001) proportions (i.e., interest areas on the *right* hemiface), of opposite sign, as more gaze in these regions was associated with a reduced likelihood of picking the left hemiface. Task did not interact with gaze to the Right Emotional hemiface (*z* = −0.20, *p* = 0.84), but there was a significant Task × Left Neutral interaction (*z* = 4.08, *p* < 0.001), as looks to the neutral side of the face affected choice behavior more in Task 2 than Task 1.

Overall, then, as expected, participants' gaze was linked to their choice behavior, as, in both tasks, they looked more at the chimeric face that they ultimately chose. However, in Task 1, there was an overall bias to look left, associated with an increased tendency to choose the left hemiface. Participants' choices were more strongly associated with gaze to the emotional halves of the faces in Task 1 compared to Task 2 but more strongly associated with gaze to the neutral halves of the faces in Task 2 relative to Task 1.

## Discussion

The aim of this study was to adjudicate between two types of accounts of the widely-documented left hemiface bias in the chimeric face test. If the observed decision bias is due to scanning patterns or attentional predispositions that are applied by default, then the pattern should hold across tasks. However, to our knowledge, no one has ever previously directly compared responses to chimeric faces while manipulating task demands. Here, therefore, we used the same stimuli and asked participants to either judge which face was more emotional (i.e., one of the tasks commonly used with chimeric faces in the literature; Task 1) or to judge which face was derived from the original photographs used to create the chimeric faces (Task 2). Critically, the subject population, stimuli, and general paradigm were all identical across the two tasks; the only difference was whether the decision that participants were asked to make would likely benefit from right hemisphere specializations for extracting emotional information from faces. In addition, we used eye tracking methods to measure gaze in order to be able to look directly at scanning biases and their relationship to the decisions that participants made.

In Task 1, we replicated findings in the chimeric face literature for all of our measures. Participants showed a robust left hemiface decision bias. We also replicated previous response time results (Bourne, 2008), in that our participants were faster to respond on trials in which they picked the chimeric face with emotion on the left than those in which they picked the face with emotion on the right. Few prior studies have measured gaze in the chimeric face task, but Butler et al. (2005) found that on trials for which participants picked the left hemiface, gaze patterns were biased, such that participants looked more at the (left) emotional side of the chosen face than the (right) emotional side of the non-chosen face. We observed an overall tendency for participants to direct gaze to the left side of the faces, consistent with the predictions of a scanning bias account, and, like Butler et al. (2005) found that an increased proportion of time spent looking at the left side of the chimeric faces was predictive of an increased left hemiface bias in choice behavior.

Of critical interest, then, was whether these same patterns would obtain when we changed the decision that participants were asked to make. In Task 2, rather than making a judgment of emotionality, participants were asked to determine which was the “original chimeric face.” The directional scanning account argues that participants have a default pattern of gaze distribution over face stimuli, which may be driven in part by reading direction in the participants' native language (Vaid and Singh, 1989)—thus, predicting a left gaze bias in our English speaking population (as was obtained in Task 1). This gaze bias, in turn, is hypothesized to drive the observed asymmetry in choice behavior in the chimeric faces task. Therefore, the directional scanning account predicts that we should observe similar gaze patterns, similar choice behaviors, and similar links between gaze and choice in both tasks, given that the stimuli and participant population were the same in both. In contrast, views that link the left hemiface bias to underlying hemispheric asymmetries in the ability to derive relevant information from the stimuli should predict a diminished or absent bias in Task 2, given that the perceptual information available from the faces does not provide a basis for judging which the original face was. (Note that because all chimeric faces were actually constructed using the left half of emotion-conveying faces, a correct answer would have yielded a left hemiface bias; our design thus provides a conservative test of the scanning bias account).

We found that all measures were notably affected by task. In striking contrast to the robust left hemiface bias observed in Task 1, participants showed no response bias at all in Task 2. Thus, participants were (as expected) not able to reliably determine which face was the original one. Moreover, participants also clearly did not just adopt a strategy of using an emotionality judgment as the basis of their judgments in Task 2, as this, too, would have yielded a pattern wherein the tasks patterned similarly. Therefore, the left hemiface bias obtained only under task conditions in which hemispheric specialization for extracting information from emotional faces could provide useful decision-related information. This pattern supports the right hemisphere specialization account of the left hemiface bias and is inconsistent with the directional scanning account.

The lateralized response time bias observed in Task 1 was also absent in Task 2. Instead, participants spent more time overall rendering a decision in Task 2 than Task 1; thus, the lack of decision bias in the second task cannot be attributed to participants “giving up” and simply guessing. These longer response times likely reflect the fact that there were no immediately obvious facial characteristics that participants could use to inform their decision, whereas participants in Task 1 were explicitly cued to examine the emotive side of the face. Thus, Task 2 participants likely distributed their search patterns more thoroughly across the two hemifaces. Indeed, Task 1 participants allocated a significantly higher proportion of their gaze to the emotional sides of the chimeric faces than did Task 2 participants.

Notably, the effect of task on gaze patterns was also inconsistent with a default scanning bias account. We did find a task-independent bias to initially (within the first 500 ms—one to at most two fixations) direct gaze to the left emotional hemiface. This is the pattern described by the default scanning bias account, possibly arising from a tendency for English readers to scan initially from left to right (e.g., Vaid and Singh, 1989). However, this pattern was short-lived and uncorrelated with later choice behavior. Participants quickly (by 1000–2000 ms) adopted and sustained task-specific gaze patterns after this initial window, which then did predict choice behavior. Whereas participants in Task 1 showed a bias to direct gaze to the left, participants in Task 2 actually showed a bias to look more at the right sides of the faces. Given that the left hemisphere has been argued to be superior in extracting local facial feature information (as opposed to more holistic/global information, which has been associated with right hemisphere face processing advantages; see, e.g., Patterson and Bradshaw, 1975; Bradshaw and Sherlock, 1982; Rossion et al., 2000), this pattern may indicate that participants made greater use of local feature information in Task 2. Irrespective of source, however, the pattern is inconsistent with the claim that the participants simply had a default bias to gaze more overall at the left side of chimeric faces.

Not only did overall gaze patterns to the faces differ across task, they were also differentially linked to choice behavior in the two tasks. The tendency to look at the left side of the face, and especially the left emotional half-face, was more predictive of a left hemiface decision bias in Task 1 than in Task 2. Moreover, gaze patterns to the neutral halves of the face were differentially linked to decision biases in the two tasks: in Task 2, relative to Task 1, participants were more likely to pick a chimeric face if they gazed longer at its neutral side.

Our study, therefore, shows for the first time that changes in task demands can have a profound impact on how participants seek information from chimeric faces and on their behavioral judgments about the faces (see Stephan et al., 2003, for analogous findings on word processing). Both individually and collectively, results from our decision and eye gaze data are more consistent with a right hemispheric specialization than a directional scanning account of the left hemiface bias in the chimeric face test. This decision bias, which is associated with faster responding as well as increased gaze to the left sides of the test faces, critically depends on the availability of useful decision-related information for which the right hemisphere has been argued to have a processing advantage (Voyer et al., 2012). That is, when emotion-related (or, in other studies, gender-related; Demaree et al., 2005; Hu et al., 2013) information in the face is useful for the decision, the better extraction of that information from the left side of the face, by the right hemisphere, induces a decision bias, which has been shown to be robust even to inversion and short presentation times (Butler and Harvey, 2005, 2006). However, when right hemisphere specialization cannot provide helpful information for the decision – as in our second task—the bias is strikingly eliminated, even with stimuli, general task demands, and participant characteristics held constant.

Finally, the observation that not only decision biases, but also gaze patterns and their relationship to participants' decisions changed with task demands, suggests that gaze, and by extension attention, may facilitate the flexible recruitment of specialized hemispheric resources. Levy and Trevarthen (1976) described what they called hemispheric “metacontrol” or the mechanisms governing which hemisphere will attempt to control information processing operations for a given task. In the chimeric faces task, Urgesi et al. (2005) have dissociated metacontrol (which hemisphere influences the response) from hemispheric specialization (right hemisphere advantages for face processing, which were seen independently of metacontrol). The present results indicate that under conditions in which the right hemisphere has an advantage for extracting decision-relevant information from a face, participants direct more gaze toward the left halves of faces. Moreover, the extent to which gaze to the left emotional hemiface predicts left hemiface bias differs across tasks and is stronger under a condition in which emotion is cued as a decision-relevant information. This pattern is consistent with Adam and Güntürkün's (2009) proposal that metacontrol might arise via a winner-takes-all type of mechanism, wherein a small initial processing advantage for one hemisphere, combined with commissural inhibition, yields unilateral dominance over the course of processing. Our data extend this proposal by suggesting a role for gaze and attention in mediating the development of this shift. Indeed, Stephan et al. (2003) found that task-related changes in processing asymmetry are associated with a corresponding change in the lateralization of control networks, including the anterior cingulated cortex, which has been linked to both attention (e.g., Weissman et al., 2004) and eye control (e.g., Paus et al., 1993). The present results suggest that gaze patterns are adapted to task demands in a manner that can facilitate hemispheric metacontrol and allow the recruitment of task-relevant asymmetric processing resources.

### Conflit of interest statement

The authors declare that the research was conducted in the absence of any commercial or financial relationships that could be construed as a potential conflict of interest.
