# Supplementary material for: Task demands modulate decision and eye movement responses in the chimeric face test: examining the right hemisphere processing account
Source: Front Psychol. 2014 Mar 20;5:229. doi: 10.3389/fpsyg.2014.00229 (PMC3960575; doi:10.3389/fpsyg.2014.00229)
Supplement: Supplementary file 1 [file DataSheet1.PDF]

Supplementary material for “Task demands modulate decision and eye movement responses in the chimeric face test: examining the right hemisphere processing account”

Jason C. Coronel[1]* and Kara D. Federmeier[2]

Correspondence:
Jason C. Coronel
University of Pennsylvania
Annenberg School for Communication
3620 Walnut Street
Philadelphia, PA 19104
jasoncoronel@gmail.com

[1]Annenberg School for Communication, University of Pennsylvania, Philadelphia, PA, USA
[2]2Department of Psychology, Program in Neurosciences, and Beckman Institute for Advanced Science and Technology, University of Illinois, Champaign, IL, USA

<u>Contents:</u>

1. Supporting Table: Influence of Task and Gaze Patterns to Four Regions of Interest on Behavioral Judgments

2. Supporting Table: Influence of Task and Early Gaze Patterns Directed to the Left Side on Behavioral Judgments

Table S1. Influence of Task and Gaze Patterns to Four Regions of Interest on Behavioral Judgments

Left Emotional

	Estimate	SE	z-value	Pr(>|z|)
Fixed Effects				
(Intercept)	2.86	.18	15.49	< .001
Task	0.89	.18	4.85	**< .001**
Left Emotional	11.07	.70	15.77	**< .001**
Task * Left Emotional	1.97	.70	2.80	**.005**

Right Emotional

	Estimate	SE	z-value	Pr(>|z|)
Fixed Effects				
(Intercept)	0.74	.12	5.99	<.001
Task	0.72	.12	5.86	**<.001**
Right Emotional	-4.97	.24	-20.68	**<.001**
Task * Right Emotional	-0.05	.24	-0.20	0.844

Left Neutral

	Estimate	SE	z-value	Pr(>|z|)
Fixed Effects				
(Intercept)	0.70	.12	5.79	<.001
Task	0.63	.12	5.26	**<.001**
Left Neutral	-2.18	.26	-8.32	**<.001**
Task * Left Neutral	1.07	.26	4.08	**<.001**

Right Neutral

	Estimate	SE	z-value	Pr(>|z|)
Fixed Effects				
(Intercept)	0.65	.12	5.17	<.001
Task	0.82	.12	6.53	**<.001**
Right Neutral	3.05	.26	11.88	**<.001**
Task * Right Neutral	-0.96	.26	-3.73	**<.001**

Table S2. Influence of Task and Early Gaze Patterns (first 0-500 time bin) to Left Side on Behavioral Judgments

	Estimate	SE	z-value	Pr(>|z|)
Fixed Effects				
(Intercept)	.69	.12	5.91	< .001
Task	.67	.11	5.81	**< .001**
Left Proportion	.04	.09	.42	.67
Task * Left Proportion	.11	.09	1.17	.24

Note: We analyzed data in a logistic mixed effects model with a binomial link function, with Task (coded as "1" for Task 1 and "-1" for Task 2), and Left Proportion (i.e., mean-centered proportion of time spent looking on the left side of the faces) as fixed effects, and participants and items as random effects. This model reveals a main effect of Task ($z = 5.81$, $p < .001$). There are no other main effects or interactions.
